# The Biological Function of DMP-1 in Osteocyte Maturation Is Mediated by Its 57-kDa C-terminal Fragment

**DOI:** 10.1002/jbmr.226

**Published:** 2010-08-23

**Authors:** Yongbo Lu, Baozhi Yuan, Chunlin Qin, Zhengguo Cao, Yixia Xie, Sarah L Dallas, Marc D McKee, Marc K Drezner, Lynda F Bonewald, Jian Q Feng

**Affiliations:** 1 Department of Biomedical Sciences, Baylor College of Dentistry, Texas A&M Health Science Center Dallas, TX, USA; 2 Department of Medicine, University of Wisconsin and GRECC, William F. Middleton Veterans Administration Hospital Madison, WI, USA; 3 Department of Oral Biology, University of Missouri–Kansas City Kansas City, MO, USA; 4 Faculty of Dentistry and Department of Anatomy and Cell Biology, McGill University Montreal, Quebec, Canada

**Keywords:** DMP-1, OSTEOCYTE MATURATION, HYPOPHOSPHATEMIC RICKETS, FGF-23, MINERALIZATION

## Abstract

Dentin matrix protein 1 (DMP-1) is a key molecule in controlling osteocyte formation and phosphate homeostasis. Based on observations that full-length DMP-1 is not found in bone, but only cleaved fragments of 37 and 57 kDa are present, and in view of the finding that mutations in the 57-kDa fragment result in disease, we hypothesized that the 57-kDa C-terminal fragment is the functional domain of DMP-1. To test this hypothesis, a 3.6-kb type I collagen promoter was used to express this 57-kDa C-terminal fragment for comparison with full-length DMP-1 in *Dmp1* null osteoblasts/osteocytes. Not only did expression of the full-length DMP-1 in bone cells fully rescue the skeletal abnormalities of *Dmp1* null mice, but the 57-kDa fragment also had similar results. This included rescue of growth plate defects, osteomalacia, abnormal osteocyte maturation, and the abnormal osteocyte lacunocanalicular system. In addition, the abnormal fibroblast growth factor 23 (FGF-23) expression in osteocytes, elevated circulating FGF-23 levels, and hypophosphatemia were rescued. These results show that the 57-kDa C-terminal fragment is the functional domain of DMP-1 that controls osteocyte maturation and phosphate metabolism. © 2011 American Society for Bone and Mineral Research.

## Introduction

Dentin matrix protein 1 (DMP-1) is expressed predominantly in odontoblasts in tooth and osteocytes in bone.(1–3) Deletion of murine *Dmp1* causes striking defects in tooth and bone during postnatal development in C57/B6 or CD-1 background mice.(4,5) Recently, we and others have demonstrated that *DMP1* mutations result in autosomal recessive hypophosphatemic rickets (ARHR) in humans similar to the phenotype of *Dmp1* null mice. This condition is characterized by rickets and the presence of large amounts of osteoid in bone (osteomalacia) and is accompanied by elevated circulating fibroblast growth factor 23 (FGF-23).(6–9) Using the *Dmp1* null mouse as a model for human ARHR, we found that *Dmp1* null osteocytes express elevated FGF-23, suggesting that *Dmp1* might be a negative regulator of FGF-23 expression during bone development.(6)

FGF-23 is a potent phosphaturic hormone expressed predominantly by osteocytes in bone(6,10–12) that target the kidney to promote renal excretion of phosphate.(13,14) In addition, *PHEX* (phosphate-regulating gene with homologies to endopeptidases on the X chromosome) also has been shown to regulate FGF-23 expression in bone.(12) *PHEX* is predominantly expressed in osteoblasts and osteocytes.(15) *PHEX* mutations in mice and humans result in autosomal dominant hypophosphatemic rickets (ADHR), accompanied by elevated circulating FGF-23, a phenotype similar to that of *Dmp1* null mice.(6,10) Taken together, these observations suggest that elevated circulating FGF-23 and hypophosphatemia are pathogenic factors involved in both *Phex* and *Dmp1* mutant mice.

We have previously identified two proteolytic fragments of DMP1, a 37-kDa N-terminal fragment and a 57-kDa C-terminal fragment, but little intact protein from rat long bone and dentin extracts.(16,17) The 57-kDa fragment is highly phosphorylated, containing 41 phosphates,(16) whereas the 37-kDa fragement was shown to be a proteoglycan with a chondroitin sulfate chain attached via Ser74.(18) These findings lead to the hypothesis that the full-length DMP-1 might represent an inactive precursor that has to be cleaved to become functional.(16) Studies using an in vitro cell-free system have indicated that full-length DMP-1, its two fragments, and the extent of their phosphorylation have distinct effects on biomineralization.(19) Specifically, the highly phosphorylated 57-kDa fragment likely functions as a hydroxyapatite nucleator.(19)

In addition, it was discovered that a *DMP1* mutation, which results in replacement of the last 18 amino acid residues with 33 novel residues, results in ARHR.(6,7) In vitro studies showed that this mutant DMP-1 is secreted and processed into the 37-kDa N-terminal fragment and the mutant 57-kDa fragment,(6) suggesting that patients should have normal 37-kDa fragment expression. These observations suggest that the 57-kDa fragment might be the key functional domain of DMP-1.

We have previously generated two separate transgenic lines of mice expressing the full-length and the 57-kDa fragment under the control of a 3.6-kb type I collagen promoter.(20) The mRNAs of both transgenes are highly expressed in osteoblasts, whereas the proteins of these transgenes are distributed predominantly in the matrix surrounding osteoblasts and osteocytes.(20) Further, mice carrying either transgene do not exhibit an apparent skeletal phenotype.(20) By crossing these transgenes onto the *Dmp1* null background, this study investigated which aspects of the *Dmp1* null skeletal phenotype could be rescued by the full-length and 57-kDa DMP-1 fragment in order to further understand the function of DMP-1 in the skeleton. Our study shows that the 57-kDa fragment recapitulates the function of full-length DMP-1 in regulation of mineralization and osteocyte maturation and highlights the molecular mechanisms responsible for hypophosphatemic rickets.

## Materials and Methods

### Mice

*Dmp1* knockout (KO) mice with a C57B/L6 genetic background have been described previously.(21) Transgenic mice overexpressing full-length DMP-1 were generated on a CD-1 genetic background,(22) and the 57-kDa C-terminal fragment transgenic mice were generated on a C57B/L6 genetic background as described previously.(20) To determine the function of the 57-kDa C-terminal fragment or the full-length of DMP-1 in vivo, these transgenes were introduced into the *Dmp1* null background by breeding the transgenic mice with *Dmp1* null mice separately. The mice were fed with autoclaved Purina rodent chow (5010, Ralston Purina, St. Louis, MO, USA) containing 1% calcium, 0.67% phosphorus, and 4.4 IU of vitamin D/g. All animal protocols were approved by the Institutional Animal Care and Use Committee.

### Quantitative real-time PCR analysis

Total RNA was extracted from 10-day-old mouse bones using Trizol reagent (Invitrogen, San Diego, CA, USA) according to the manufacturer's protocol. One microgram of total RNA then was treated with Turbo DNase (Ambion, Austin, TX, USA) and reverse transcribed into first-strand cDNA using a high-capacity cDNA reverse-transcription kit (Invitrogen). Quantitative real-time PCR was performed using SYBR Green PCR mix plus gene-specific primers in a 7700 Sequence Detection System (Applied Biosystems, Foster City, CA, USA). PCR conditions were as follows: 40 cycles of 95°C for 15 seconds, 60°C for 30 seconds, and 72°C for 30 seconds. For each gene, three independent PCRs from the same reverse-transcription sample were performed. The presence of a single specific PCR product was verified by melting-curve analysis, confirmed on an agarose gel, and further sequenced by the Applied Biosystems sequencing system (Applied Biosystems). Genes were analyzed including *osteocalcin* (*OCN*), *alkaline phosphatase* (*ALP*), *MEPE*, and *sFRP4*. *GAPDH* was used as an internal control. The primer sequences used are *OCN* forward primer (F), 5”-CTC TGT CTC TCT GAC CTC ACA G-3”, and reverse primer, 5'V-GGA GCT GCT GTG ACA TCC ATA C-3”; *ALP* forward primer, 5'-CTT GCT GGT GGA AGG AGG CAG G-3”, and reverse primer, 5”-CAC GTC TTC TCC ACC GTG GGT C-3”; *MEPE* forward primer: 5”-TGT TGG ACT GCT CCT CTT CA-3”, and reverse primer, 5”-TCC TTC CCT CCG TAG TTC G-3”; *sFRP4* forward primer, 5”-GTG CAA GTG CAA AAA GGT GA-3”, and reverse primer, 5”-ATC ATC CTT GAA CGC CAC TC-3”; and *GAPDH* forward primer, 5”-GGT GTG AAC CAC GAG AAA-3”, and reverse primer, 5”-TGA AGT CGC AGG AGA CAA-3”.

### Fluorochrome labeling of the mineralization front

To visualize bone mineralization in mice, double fluorescence labeling was performed as described previously.(22) Briefly, mice were first injected intraperitoneally with calcein green (5 mg/kg), followed by injection of an alizarin red label (5 mg/kg i.p.; Sigma-Aldrich, St Louis, MO, USA) 5 days later. Mice were euthanized 48 hours after injection of the second label, and the bones were removed and fixed in 70% ethanol for 48 hours. The specimens were dehydrated through a graded series of ethanol (70% to 100%) and embedded in methyl methacrylate (MMA) without prior decalcification. The 50-mm nondecalcified samples from these animals were photographed using a Nikon PCM-2000 confocal microscope coupled with an Eclipse E-800 upright microscope (Nikon Instruments, Melville, NY, USA) for fluorochrome labeling or in combination with 4,6-diamidino-2-phenylindole (DAPI) staining of nuclei of osteocytes.

### Visualization of the lacunocanalicular system by procion red

This small molecular dye was injected through the tail vein (0.8%, 0.01 mL/g) under anesthesia with 2,2,2-Tribromoethanol (5 mg/kg of body weight) 10 minutes before euthanization. The fresh bone was fixed in 70% EtOH and sectioned at 50 µm thickness using a Leitz 1600 saw microtome (Ernst. Leitz Wetzlar GmbH, Wetzlar, Germany). Sections were imaged using a Nikon C100 confocal microscope with an optronics cooled CCD (charge-coupled device) camera.

### Scanning electron microscopy (SEM) and transmission electron microscopy (TEM)

To image the osteocyte lacunocanalicular system, SEM of resin-casted bone samples was performed. Bone tissues were fixed in 70% ethanol and embedded in MMA (Buehler, Lake Bluff, IL, USA). The surface of the MMA-embedded bone was polished, followed by acid etching with 37% phosphoric acid for 2 to 10 seconds, 5% sodium hypochlorite for 5 minutes, and coating with gold and palladium. Samples were examined by an FEI/Philips XL30 field emission environmental SEM (Phillips, Hillsboro, OR, USA). For TEM, specimens were fixed with 4% paraformaldehyde and 1% glutaraldehyde in 0.1 M sodium cacodylate buffer (pH 7.2), then postfixed with 1% osmium tetroxide in 0.1 M sodium cacodylate buffer, and processed for embedding in LR White acrylic resin (London Resin Company, Bershire, UK). Colloidal-gold immunostaining of DMP-1 was performed and examined by TEM using a JEOL 2000FXII transmission electron microscope (Jeol Ltd., Tokyo, Japan) as described previously.(22)

### Radiography and micro–computed tomography (µCT)

The long bones or vertebrae were dissected and X-rayed using a Faxitron radiographic inspection unit (Model 8050-020, Field Emission Corporation, Inc., McMinnville, OR, USA) with digital image capture capabilities. The long bones, vertebrae, and metatarsals were scanned in a µCT imaging system (35, Scanco Medical, Bassersdorf, Switzerland) as described previously.(4,5)

### Histology

For paraffin block preparations, specimens were fixed in freshly prepared 4% paraformaldehyde in PBS (pH 7.4), decalcified, and embedded in paraffin by standard histological procedures, as described previously.(3) Then 5-µm sections were cut and dried. Sections were used for Safranin-O staining, immunohistochemistry, TRAP staining, and in situ hybridization using digoxigenin-labeled antisense RNA (cRNA) probes as described previously.(6) For undecalcified bones, specimens were embedded in MMA and cut at 6-µm thickness using a Leica 2165 rotary microtome (Ernst. Leitz Wetzlar GmbH). Undecalcified sections were stained by von Kossa staining,(21) Goldner's Masson trichrome staining.(6) alizarin red/alcian blue staining,(21) and alkaline phosphatase (ALP) cytochemistry.(23)

### Serum biochemistry

Serum calcium, phosphorus, and FGF-23 levels were determined as described previously.(6) Briefly, serum calcium content was analyzed using a colorimetric calcium kit (Stanbio Laboratory, Boerne, TX, USA). Serum phosphorus was measured by the phosphomolybdate–ascorbic acid method. Serum FGF-23 level was determined by a full-length FGF-23 ELISA kit (Kainos Laboratories, Tokyo, Japan).

### Statistical analysis

Data analysis was performed with Student's *t* tests for two-group comparison and with one-way ANOVA for multiple-group comparisons. If significant differences were found with one-way ANOVA, the Bonferroni method was used to determine which groups were significantly different from others. The quantified results are represented as the ± standard error of the mean (SEM). *p* < .05 was considered statistically significant.

## Results

### Targeted expression of DMP-1 in osteoblasts/osteocytes but not in chondrocytes

**Fig. 1:**
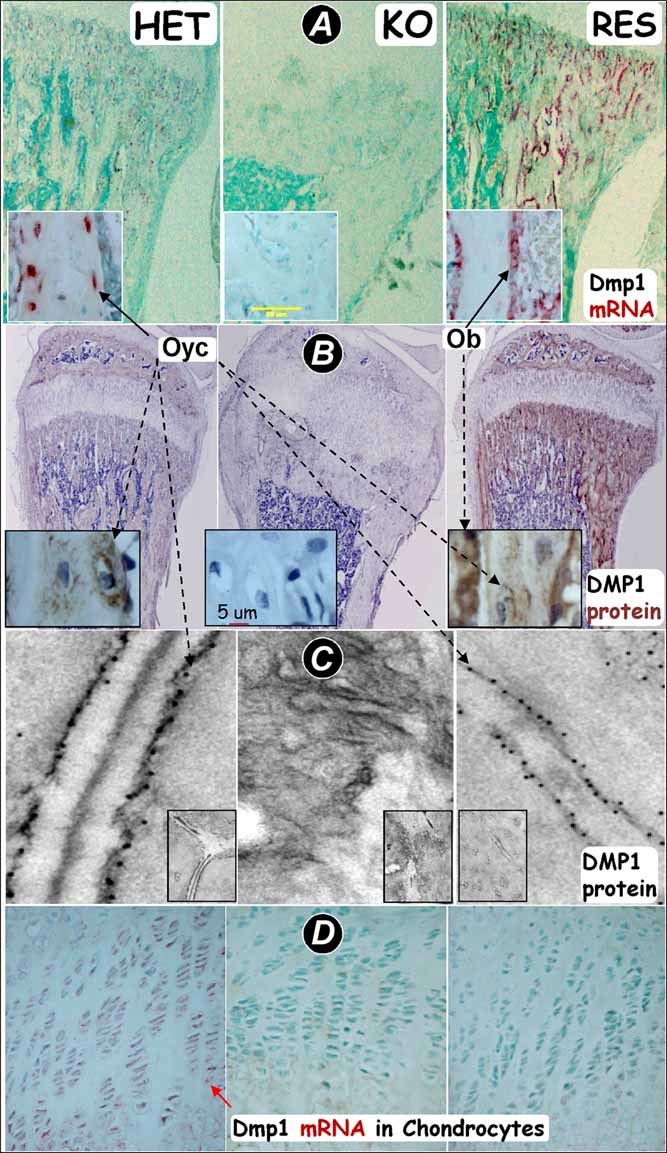
Reexpression patterns of full-length DMP-1 in *Dmp1* null (KO) mice driven by the 3.6-kb murine Col1a1 promoter. DMP-1 expression in *Dmp1* heterozygous (HET) control mice (*left panels*), *Dmp1* null mice (*middle panels*), and *Col1a1* full-length DMP-1 in the *Dmp1* null background (RES, *right panels*) was analyzed in tibias from 4-week-old mice by in situ hybridization (*a*, signal in red color), immunohistochemistry (*b*, signal in brown color), and immunogold labeling and transmission electron microscopy (*c*). The assays showed that expression of endogenous DMP-1 in HET mice was found predominantly in the osteocytes (Oyc, *black arrows*) embedded in the bone matrix. No expression of endogenous DMP-1 was detected in *Dmp1* null mice. Note that in situ hybridization showed that the transgene was highly expressed in osteoblasts (Ob, *black arrows*) but that the protein is present in both osteoblasts and osteocytes. Immunogold staining showed that the endogenous DMP-1 protein was localized to the lamina limitans at the edges of the canaliculi in the bone matrix in HET mice. As expected, there was no DMP-1 protein present in the *Dmp1* null mouse bone (*middle panels* in *a*, *b*, and *c*). However, the transgenic DMP-1 protein was localized predominantly in both the osteoblast layer and in lamellae closely adjacent to the osteocytes (RES). Note that in *d*, the endogenous *Dmp1* mRNA was weakly expressed in chondrocytes (*left panel*), but the transgenic *Dmp1* mRNA was not detected in chondrocytes (*right panel*).

Although DMP-1 is highly expressed in osteocytes,(24) DMP-1 is also expressed to a much lower extent in osteoblasts, particularly during embryonic development.(21,25) To better understand the functions of DMP-1 in bone, the murine 3.6-kb Col1a1 promoter was used to drive *Dmp1* transgenes. Figure 1*A* shows that the full-length *Dmp1* mRNA was targeted to osteoblasts, but the protein was highly expressed in both osteoblasts and osteocytes to a much higher level than endogenous DMP-1 (Fig. 1*B*, *right panel*). Importantly, immunogold TEM data showed the same distribution pattern of both transgenic and endogenous DMP-1 protein in the lamina limitans of the extracellular matrix immediately surrounding osteocyte processes in the HET control mice, no DMP-1 in the *Dmp1* null mice, and restoration of DMP-1 protein in *Dmp1* null osteocytes (Fig. 1*C*). By in situ hybridization, note that DMP-1 is weakly expressed in chondrocytes, but there was no signal in *Dmp1* null or the rescued chondrocytes (Fig. 1*D*).

### Reexpression of full-length DMP-1 rescued the skeletal abnormalities of *Dmp1* null mice

The full-length *Dmp1* transgene was introduced into the *Dmp1* null background mice by crossing full-length *Dmp1* transgenic mice with *Dmp1* null mice. Multiple evaluations then were used to examine whether reexpression of the full-length DMP-1 could rescue the *Dmp1* null phenotype.

**Fig. 2:**
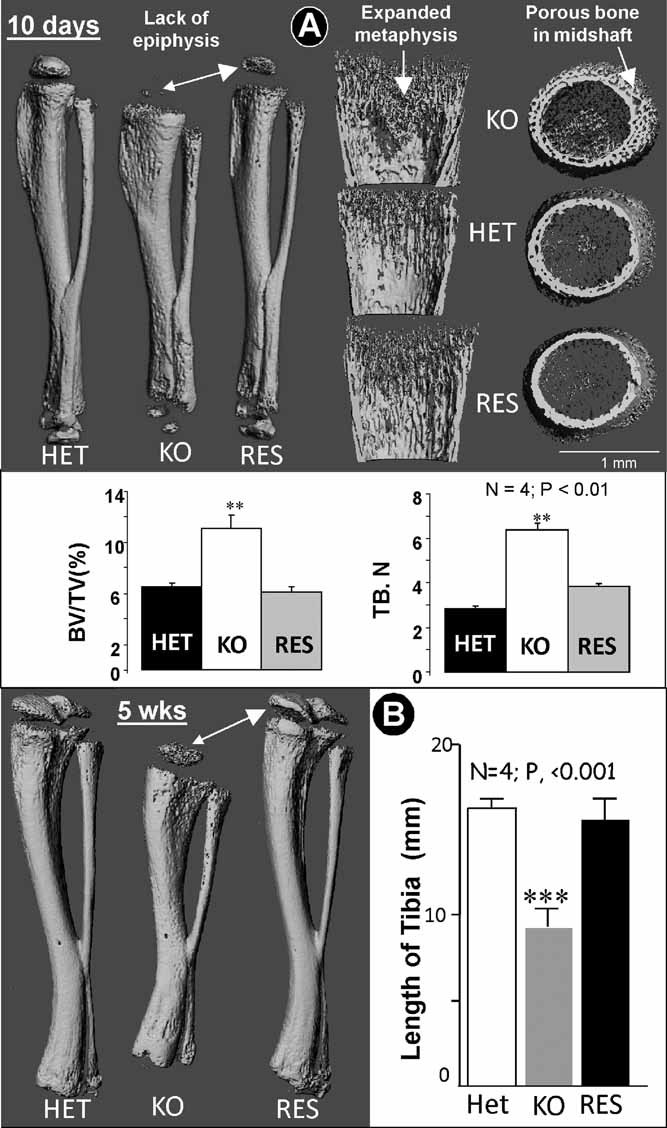
Rescue of *Dmp1* null bone abnormalities with the full-length *Dmp1* transgene at the ages of 10 days and 5 weeks. (*A)* Representative µCT images of tibias from the HET, KO, and rescued mice (RES) at the age of 10 days. The whole-mount view is shown in the left panel, the sagittal sections of metaphyses are shown in the middle panel, and the cross sections of midshaft are shown in the right panel; the quantified data are shown in the lower panels. (Data are mean ± SEM; *n* = 4; ^***^p < .001). (*B*) Whole-mount views of 5-week-old tibias from the HET, KO, and rescued (RES) mice are shown in the left panel; the quantified data are shown in the right panel. (Data are mean ± SEM; *n* = 4; ^***^p < .001).

At day 10, ossification in *Dmp1* null epiphyses was largely undetectable by µCT (*arrow* in Fig. 2*A*, *left panel*), but the *Dmp1* null epiphyses were partially restored when full-length DMP-1 was reexpressed in bone. Second, the porous nature of the *Dmp1* null and expanded metaphyses was fully rescued, as documented by both representative µCT images and quantified data (Fig. 2*A*, *right* and *lower panels*). Note that at the age of 5 weeks, the *Dmp1* null epiphysis and the bone length were fully rescued by the full-length DMP-1 (Fig. 2*B*). This result indicates that there is a delay in the rescue of *Dmp1* null epiphysis phenotype at an early stage, but this phenotype is fully rescued at 5 weeks of age.

### Reexpression of full-length DMP-1 rescued mineralization abnormalities of *Dmp1* null mice

**Fig. 3:**
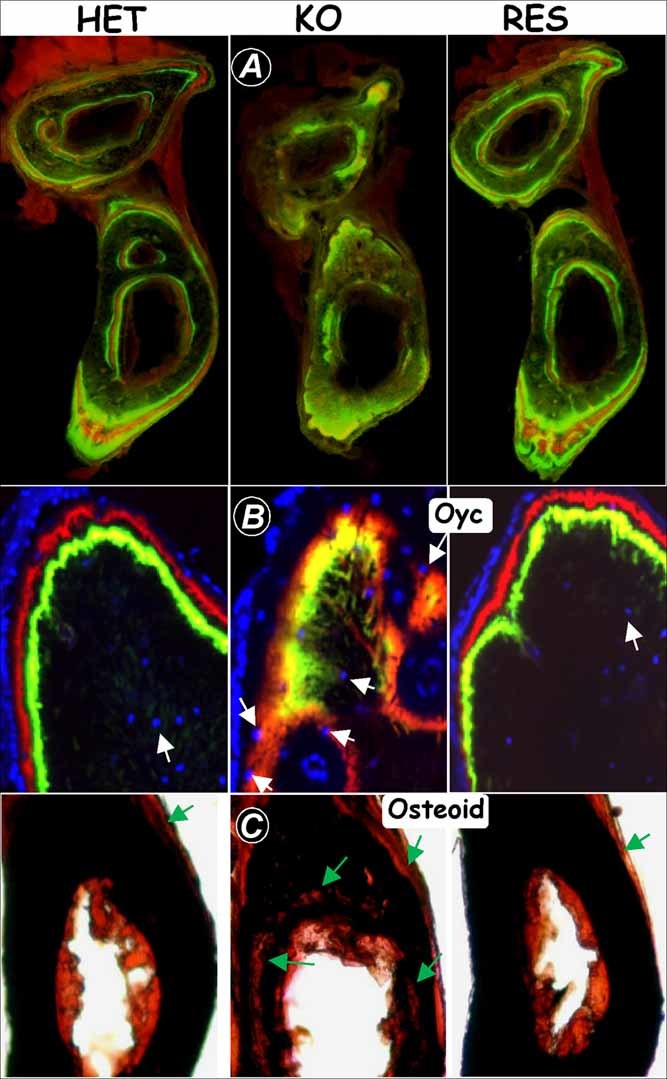
Rescue of *Dmp1* null bone defects in mineralization with the full-length *Dmp1* transgene. (*A*) The double fluorochrome labels displayed an identical bone-formation rate in HET (*left panel*) and RES (*right panel*) mice in contrast to the diffuse label in KO mice (*middle panel*) at 2 months of age. Diffuse fluorochrome labels in the *Dmp1* null mice (*middle panel*) were fully restored in the rescue group (*right panel*). (*B*) Confocal microscopic images of fluorochrome labeling, counterstained with DAPI for visualization of osteocyte nuclei (*blue*, *white arrows*), showed that osteocytes are separated from the mineralization front in HET control mice (*left panel*) and RES mice (*right panel*). Diffuse fluorochrome labeling in the *Dmp1* null mice (*middle panel*) was replaced with sharp lines in the RES group (*right panel*). (*C*) von Kossa staining showed restored mineralization in the RES mice, whereas abundant osteoid (*red color*, *arrows*) was present in the KO tibias at the age of 2 months.

One of the key defects in *Dmp1* null mice is defective mineralization.(4,6) Using a fluorochrome labeling technique, three discrete label lines were observed in both the control and rescued mice in contrast to the *Dmp1* null mice (Fig. 3*A*). Similarly, the images combining fluorochrome labeling with DAPI nuclear staining, used to visualize the position of osteocytes relative to mineralization fronts, showed a complete rescue by the full-length DMP-1 (Fig. 3*B*). The von Kossa stain, a common staining protocol used for detection of mineralization defects, showed a complete rescue of the osteomalacia in *Dmp1* null mice by the full-length *Dmp1* transgene (Fig. 3*C*). Taken together, these results demonstrate that the full-length DMP-1 completely rescues mineralization defects observed in *Dmp1* null long bone.

### Reexpression of full-length DMP-1 rescued the defects in the osteocyte lacunocanalicular network and in the growth plate

**Fig. 4:**
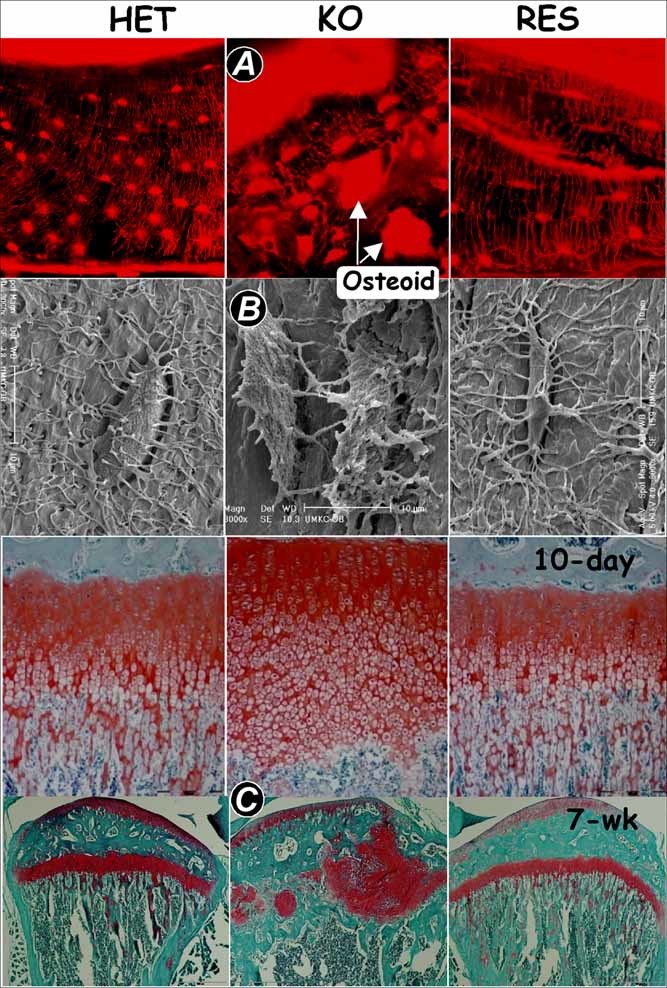
Rescue of the malformed osteocyte lacunocanalicular network and growth plate defects. (*A*) Visualization of the disorganized osteocyte canalicular system in 7-week-old *Dmp1* null mice (*middle panel*) with procion red injection compared with the well-organized control osteocytes (*left panel*) and the rescued osteocytes (*right panel*) using confocal microscopy at 565-nm excitation and 610-nm emission. (*B*) SEM images of the acid-etched, resin-casted osteocyte canalicular system from the 7-week-old HET control mice (*left panel*), the *Dmp1* null mice (*middle panel*), and the rescued mice(*right panel*). Note that the poorly formed osteocyte canalicular system in *Dmp1* null mice was fully rescued by the full-length *Dmp1* transgene. (*C*) Safranin-O staining of the tibia growth plate, showing that *Dmp1* null mice displayed about a threefold increase in the hypertrophic zone at the age of 10 days and formation of multiple chondrocyte clusters with a loss of continuity at 7 weeks of age (*middle panels*). All these morphological changes were completely rescued by the full-length *Dmp1* transgene in the *Dmp1* null background (*right panels*) compared with the HET controls (*left panels*).

Osteocytes and their dendrites are located in the porosities in bone, the lacunocanalicular network. In *Dmp1*-HET control mice, this system is revealed by the fluorescent images of bone sections after injection of a small molecular dye, procion red (Fig. 4*A*, *left panel*), and the smooth walls of the lacunae and canaliculi by SEM of acid-etched, resin-casted bone samples (Fig. 4*B*, *left panel*). These methods also documented the abnormal lacunocanalicular system in *Dmp1* knockout (KO) bone showing a reduction in the number of canaliculi and expanded osteocyte lacunae with rough, not smooth, lacunar walls (Fig. 4*A, B*, *middle panels*), which was completely rescued by reexpression of full-length DMP-1 (Fig. 4*A, B*, *right panels*). Taken together, these observations support a direct role of DMP-1 in maintaining the osteocyte lacunocanalicular system.

Even though DMP-1 is weakly expressed in chondrocytes (Fig. 1*D*),(26) the *Dmp1* null rickets phenotype is likely attributable to hypophosphatemia.(6) Here we show that the hypertrophic chondrocyte zone in *Dmp1* null mice is remarkably expanded with few changes in other growth plate zones at the age of 10 days, as revealed by Safranin O staining (Fig. 4*C*, *upper middle panel*). At the age of 7 weeks, the *Dmp1* null growth plate lost its continuity and formed multiple chondrogenic clusters of different sizes (Fig. 4*C*, *lower middle panel*). Reexpression of full-length DMP-1 rescued essentially all these defects in the *Dmp1* null growth plates (Fig. 4*C*, *right panels*), which are identical to the age-matched controls (Fig. 4*C*, *left panels*). These data support an indirect role of DMP-1 in chondrogenesis because the *DMP1* transgene is not expressed in chondrocytes (Fig. 1*D*)

### Reexpression of full-length DMP-1 restored the gene expression profile of the *Dmp1* null bone

**Fig. 5:**
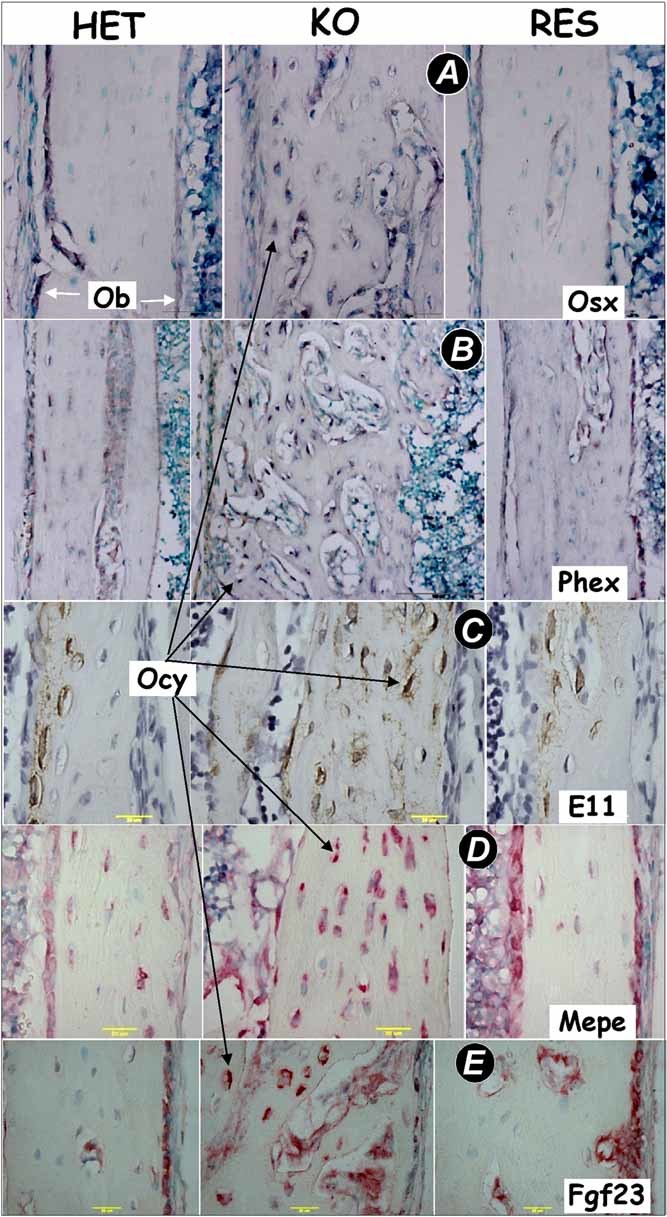
Targeted expression of DMP-1 rescued the gene expression patterns in the *Dmp1* null cortical bone. In situ hybridization (*A*, *Osx*, osterix; *B*, *Phex*, phosphate-regulating gene with homologies to endopeptidases on the X chromosome; *D*, *MEPE*, matrix extracellular phosphoglycoprotein; *E*, *Fgf23*, fibroblast growth factor 23) and immunohistochemistry (*C*, E11) assays were performed on the HET control tibias (*left panels*), the *Dmp1* null tibias (*middle panels*), and the RES (*right panels*) tibias at the age of 10 days. All these genes in *Dmp1* null cortical bone were increased (mainly in osteocytes) and were restored to control levels by targeted expression of the full-length *Dmp1*. Signal in dark purple color (*A*, *B*), in brown color (*C*), and in red color (*D*, *E*).

Our previous study(6) has shown that *Dmp1* null osteoblasts/osteocytes have altered functions. The osteocytes continuously express some osteoblast/early osteocyte–related genes, such as type I collagen (a marker for osteoblasts), E11/gp38 (a marker for early osteocytes), and FGF-23, and fail to differentiate into mature osteocytes. In this study, we screened 13 molecular markers, including Osx (osterix, a transcriptional factor essential for bone formation, mainly expressed in osteoblasts), Phex (its mutations lead to a phenotype very similar to that seen with *Dmp1* mutations), E-11/gp38, MEPE (matrix extracellular phosphoglycoprotein, expressed in both osteoblasts and osteocytes), and FGF23 (Fig. 5) as well as five common osteoblast markers, osteocalcin (OC), bone sialoprotein (BSP), osteopontin (OPN), biglycan, and Col I (type I collagen) in two long bone sites: cortical bone and trabecular bone (Supplemental Fig S1). All these markers were increased dramatically in *Dmp1* null osteocytes (Fig. 5 and Supplemental Fig. S1, *middle panels*), and reexpression of full-length DMP-1 restored their normal expression patterns (Fig. 5 and Supplemental Fig. S1, *right panels*), suggesting that the delay in osteocyte maturation was rescued. This in vivo rescue confirms that DMP-1 is essential for osteocyte maturation and normal bone mineralization. The full-length DMP reexpression model serves as an ideal control for determining the in vivo biological function of the 57-kDa C-terminal fragment.

### The 57-kDa C-terminal fragment of DMP-1 restores the *Dmp1* KO phenotype to normal

**Fig. 6:**
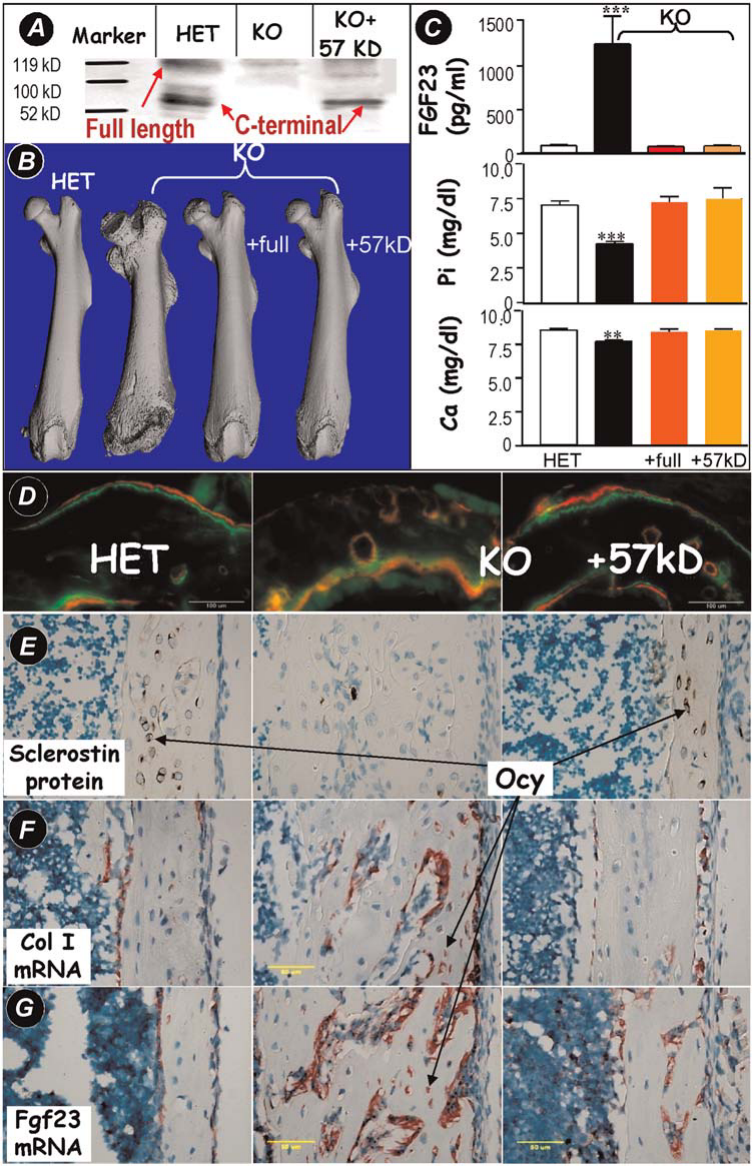
Targeted expression of the full-length DMP-1 or the 57-kD C-terminal fragment in *Dmp1* KO mice rescued the skeletal abnormalities, serum FGF-23, P_*i*_ homeostasis, and malformed osteocyte lacunocanalicular network. (*A*) Western blot data obtained from HET, KO, and rescued long bones documenting expression of the 57-kDa fragment in 2-month-old *Dmp1* KO mice. (*B*) µCT images showing similar restoration of the morphology of the femur by targeted expression of either the full-length DMP-1 or the 57-kDa fragment in the *Dmp1* KO background at 7 weeks of age. (*C*) Restoration of serum levels of FGF-23 (*upper panel*), P_*i*_ (phosphorus, *middle panel*), and Ca (calcium, *lower panel*) to normal levels in both the full-length and the 57-kDa rescued mice at 7 weeks of age; Data are mean ± SEM; *n* = 4 to 6; ^**^p < .01; ^***^p < .001. (*D*) The double fluorochrome labels showed a similar bone-formation rate in HET mice (*left panel*) and the 57-kDa RES mice (*right panel*) in contrast to the diffuse label in the KO mice (*middle panel*) at 7 weeks of age. Immunohistochemistry (*E*, sclerostin) and in situ hybridization (*F*, type I collagen; *G*, FGF-23) assays were performed on HET control tibias (*left panels*), *Dmp1* KO tibias (*middle panels*), and the 57-kDa RES tibias (*right panels*) at the age of 10 days. Sclerostin, a marker for mature osteocytes, in *Dmp1* KO osteocytes was largely undetectable, whereas *ColI* and *Fgf23* mRNAs were increased dramatically in the *Dmp1* KO osteocytes. All these changes were restored to control levels by targeted expression of the 57-kDa C-terminal fragment. Signal in brown color (*E*) and in red color (*F*, *G*).

Having established that reexpression of full-length DMP-1 rescued the skeletal abnormalities of *Dmp1* null mice, we next examined the in vivo function of the 57-kDa C-terminal fragment. Using the same strategy, the 57-kDa fragment was reexpressed in *Dmp1* null osteoblasts/osteocytes by crossing Col1a1-57K transgenic mice with *Dmp1* null mice. Western blotting analysis confirmed reexpression of the 57-kDa C-terminal fragment in the *Dmp1* KO bones (Fig. 6*A*). Similar to the full-length DMP-1, targeted reexpression of the 57-kDa C-terminal fragment in the *Dmp1* null mice rescued the bone morphology (Fig. 6*B*), the serum levels of FGF-23 (Fig. 6*C*, *upper panel*), P_*i*_ (Fig. 6*C*, *middle panel*), and Ca (Fig. 6*C*, *lower panel*), the bone-formation rate (Fig. 6*D*, *right panel*) as well as the key markers such as sclerostin (Fig. 6*E*, *right panel*), type I collagen (Fig. 6*F*, *right panel*), and FGF-23 (Fig. 6*G*, *right panel*). Finally, quantitative real-time PCR analysis showed that OCN, ALP, MEPE, and sFRP-4 are elevated in *Dmp1* KO mice and that their expression levels were restored to the control levels by reexpression of the 57-kDa C-terminal fragment (Table 1).

**Table 1:** Quantitative Analysis of the Expression of Bone Marker Genes in Various Genotypes

	Full length DMP1				57 KD			
		
Gene	HET	KO	RES	*p* value	HET	KO	RES	*p* value
OCN	1.12 ± 0.25	3.167 ± 0.29	0.797 ± 0.175	<0.05	1.016 ± 0.095	1.827 ± 0.29	1.089 ± 0.202	<0.05
ALP	1.038 ± 0.143	2.21 ± 0.215	1.146 ± 0.29	<0.05	1.091 ± 0.245	1.813 ± 0.179	0.757 ± 0.116	<0.05
MEPE	1.038 ± 0.135	7.37 ± 4.3	0.58 ± 0.26	<0.05	1.025 ± 0.129	2.745 ± 0.5	0.96 ± 0.167	<0.05
sFRP-4	1.02 ± 0.232	5.356 ± 2.23	1.545 ± 0.41	<0.05	1.04 ± 0.2	1.567 ± 0.335	0.579 ± 0.115	<0.05

Data shown are means ± SEM from four 10-day-old mice. Data analyzed by One-way ANOVA.

Taken together, these data show that the DMP-1 57-kDa C-terminal fragment possesses the biological function of the intact protein to promote osteocyte maturation and maintenance of phosphate homeostasis.

## Discussion

DMP-1, an extracellular matrix protein highly expressed in osteocytes, is processed into a 37-kDa N-terminal fragment and a 57-kDa C-terminal fragment(16); however, it was not known which fragment is essential for DMP-1 function. In this study we used targeted reexpression of full-length DMP-1 or its 57-kDa C-terminal fragment in *Dmp1* null osteoblasts/osteocytes to investigate the specific roles of the C-terminal fragment relative to the full-length DMP-1 in vivo. Our data show that both transgenes fully rescue the skeletal abnormalities of *Dmp1* null mice as well as normalizing elevated circulating FGF-23 levels and hypophosphatemia. These results support the hypothesis that the 57-kDa C-terminal fragment is responsible for the biological function of DMP-1 in maintaining phosphate homeostasis through controlling *Fgf23* expression in bone. In addition, our study supports the concept that DMP-1 controls osteocyte maturation through downregulation of many osteoblast-specific genes, such as *Osx* and *Col1*, as well as upregulation of the osteocyte-specific gene (*Sost*).

### The 57-kDa C-terminal fragment is the biologically functional domain of DMP-1

We reported previously that DMP-1 was cleaved into a 37-kDa N-terminal fragment and a 57-kDa C-terminal fragment found in the extracellular matrix of bone and dentin.(16) This suggests that full-length DMP-1 may not be an active molecule but is most likely an inactive precursor.(27) The enzyme responsible for processing DMP-1 was identified subsequently as bone morphogenetic protein 1 (BMP-1)/tolloid-like metalloproteinase.(28) This group of enzymes is not tissue-specific because they are widely expressed in many mesenchymally derived tissues and have been shown to cleave several other protein precursors, including those of several collagens (types I, II, III, V, VII, and VI), biglycan, and lysyl oxidase at selected X-Asp bonds.(24)

Mutations identified in the human *DMP1* gene that are responsible for ARHR phenotype include (1) p.M1V, (2) c.1483del1490, removing the last 18 amino acid residues, and (3) deletion of 49 kb between *DMP1* exon 3 to an intergenic region 5' to integrin-binding sialoprotein.(6,9,29) The first missense mutation of the signal peptide results in abnormal targeting to the cytoplasm, and the third mutation is a functionally null allele with no DMP-1 expression. Interestingly, protein transcribed from the second mutation, c.1483del1490, where the C-terminal 18 amino acid residues are replaced with 33 novel amino acid residues, can be transported properly to the trans-Golgi network and is processed properly to produce a normal 37-kDa N-terminal fragment but a mutant 57-kDa C-terminal fragment. The fact that these patients have an ARHR phenotype underscores the importance of the C-terminal portion of the molecule in DMP-1 function. Together, these human genetic studies and our in vivo data support the conclusion that the 57-kDa fragment is the functional domain of DMP-1.

The normal 57-kDa fragment also contains the RGD (Arginine-Glycine-Aspartic Acid) integrin-binding sequence,(30) a nuclear localization sequence,(31) and the minhibin ASARM (acidic serine- and aspartate-rich motif) peptide shown to inhibit mineralization.(32,33) Therefore, future studies are needed to determine the contributions of these functional domains to the biological activity of DMP-1 and to further refine the functional domains of the 57-kDa fragment. These findings could aid in the development of synthetic peptides or small molecules mimicking DMP-1 for the treatment of bone diseases such as hypophosphatemic rickets. At this time, the role of the 37-kDa fragment in bone biology is not clear and remains to be tested in vivo.

Similar to the *Dmp1* KO phenotype, an identical phenotype has been observed in *Hyp* mice, which carry mutations in the *Phex* gene, as well as in patients with *PHEX* mutations. The overlap in phenotypes with *PHEX* and *DMP1* loss of function, together with the full rescue of the *Dmp1* null phenotype by the 57-kDa C-terminal fragment, is suggestive that DMP-1 may be processed by PHEX. However, studies from our lab as well as others do not support such a role for PHEX.(7,20) It is also of note that the expression of DMP-1 does not appear to be altered in *Hyp* mice (data not shown). More interestingly, in contrast to the full rescue of the *Dmp1* KO phenotype by DMP-1 reexpression, the targeted reexpression of *Phex* in *Hyp* mice failed to rescue most of the phenotype, as reported by three independent research groups.(34–36) The promoters that have been used to drive the wild-type *Phex* gene include osteocalcin, the 2.3-kb Col1A1, two bone-specific promoters, and the ubiquitous human β-actin promoter.(34–36) Although the underlying reason is unknown, it has been suggested that the mutated PHEX protein acts in a dominant-negative manner and interferes with the function of the native protein. Future studies are needed to further elucidate the relationship of DMP-1 and PHEX in regulation of FGF-23 and phosphate homeostasis.

### DMP has no direct role in the growth plate

We have shown previously that deletion of *Dmp1* led to severe growth plate defects, which were thought to be due to a reduction in apoptosis in chondrogenesis.(4) A high-phosphate diet improved the defects dramatically in the growth plate of *Dmp1* null mice,(6) suggesting an indirect effect of DMP-1 on the growth plate through phosphate regulation. Interestingly, Maciejewska and colleagues(37) showed that DMP-1 is indeed expressed in chondrocytes, suggesting that DMP-1 may have both direct and indirect roles in control of chondrogenesis. To better address this issue, we used the 3.6-kb Col1a1 promoter to target the *Dmp1* transgene only in bone but not cartilage. Our in situ hybridization data confirmed that there was no expression of the transgene in the cartilage. However, the *Dmp1* null growth plate phenotype was fully rescued, which is in agreement with an indirect effect of DMP-1 on the growth plate through FGF-23 expression in bone to regulate phosphate homeostasis.(6) Thus we conclude that the *Dmp1* null growth plate phenotype is attributable to hypophosphatemia.

### DMP is a key molecule that controls osteocyte maturation

Unlike maturation and matrix production by osteoblasts characterized by increases in gene expression and cell metabolic activity, there is a dramatic decrease in gene expression and metabolic activity during maturation of osteocytes. Our previous studies(6) and current findings suggest that DMP-1 is an ideal molecule to control osteocyte maturation. The supporting evidence includes (1) DMP-1 is highly expressed in osteocytes with a very low level of expression in osteoblasts, (2) *Dmp1* null mice display significant pathological changes in the osteocyte lacunocanalicular system, (3) many genes expressed in normal osteoblasts either remained active in *Dmp1* null osteocytes (such as type I collagen or BSP) or were ectopically produced (such as FGF-23 and osteocalcin), and (4) the preceding abnormalities are rescued by targeted expression of either the full-length of DMP-1 or the 57-kDa fragment.

**Fig. 7:**
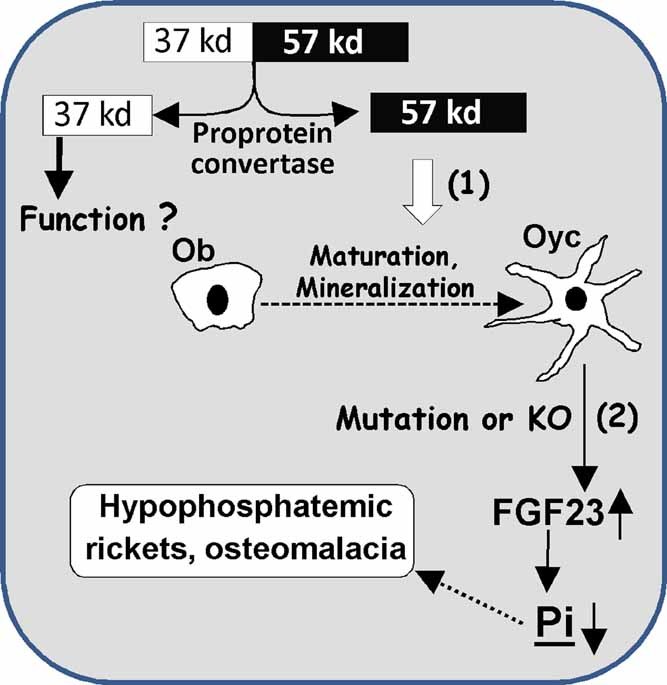
A working model of DMP-1 function in osteocyte maturation, mineralization, and phosphate homeostasis. We propose that the full-length DMP-1, secreted mainly from osteocytes (Oyc), is cleaved by BMP-1/subtilisin-like proprotein convertase into a 37-kDa N-terminal fragment and a 57-kDa C-terminal fragment.(16,24) The function of the former is not known, whereas the latter (57-kDa C-terminal) (1) likely accelerates osteocyte maturation through downregulation of osteoblast-expressed genes, such as *Osx* and *Col1*, and mineralization, and (2), FGF-23, which is normally secreted from osteoblasts (Ob) but sharply increased in osteocytes in diseases such as *Phex* mutations or *Dmp1* mutations or these knockout mouse models, decreases phosphorus (P_*i*_) reabsorption in kidney, leading to hypophosphatemic rickets and osteomalacia.

Taken together, we propose the following working model for DMP-1 (Fig. 7). DMP-1 is processed into a 37-kDa N-terminal fragment and a 57-kDa C-terminal fragment by BMP-1 or a subtilisin-like proprotein convertase.(16,20,28) The 57-kDa C-terminal fragment is the functional domain of DMP-1, controlling osteocyte maturation and mineralization. The 57-kDa C-terminal fragment also plays a key role in maintaining phosphate homeostasis through regulating *Fgf23* expression in bone. The 37-kDa N-terminal fragment is a proteoglycan.(18) It may function as a propeptide but has little, if any, role in osteocyte maturation and bone mineralization. This is supported by the observation that human patients with *DMP1* mutations in the C-terminal end of DMP-1 still suffer from ARHR even though they have normal 37-kDa N-terminal expression.(6) Our current findings suggest that small synthetic peptides in the C-terminus of DMP-1 (such as ASARM) could be developed for clinical treatment of hypophosphatemic disorders in the future.
