# Supplementary material for: The Biological Function of DMP-1 in Osteocyte Maturation Is Mediated by Its 57-kDa C-terminal Fragment
Source: J Bone Miner Res. 2010 Aug 23;26(2):331–40. doi: 10.1002/jbmr.226 (PMC3179348; doi:10.1002/jbmr.226)
Supplement: Supplementary file 1 [file jbmr0026-0331-SD1.pdf]

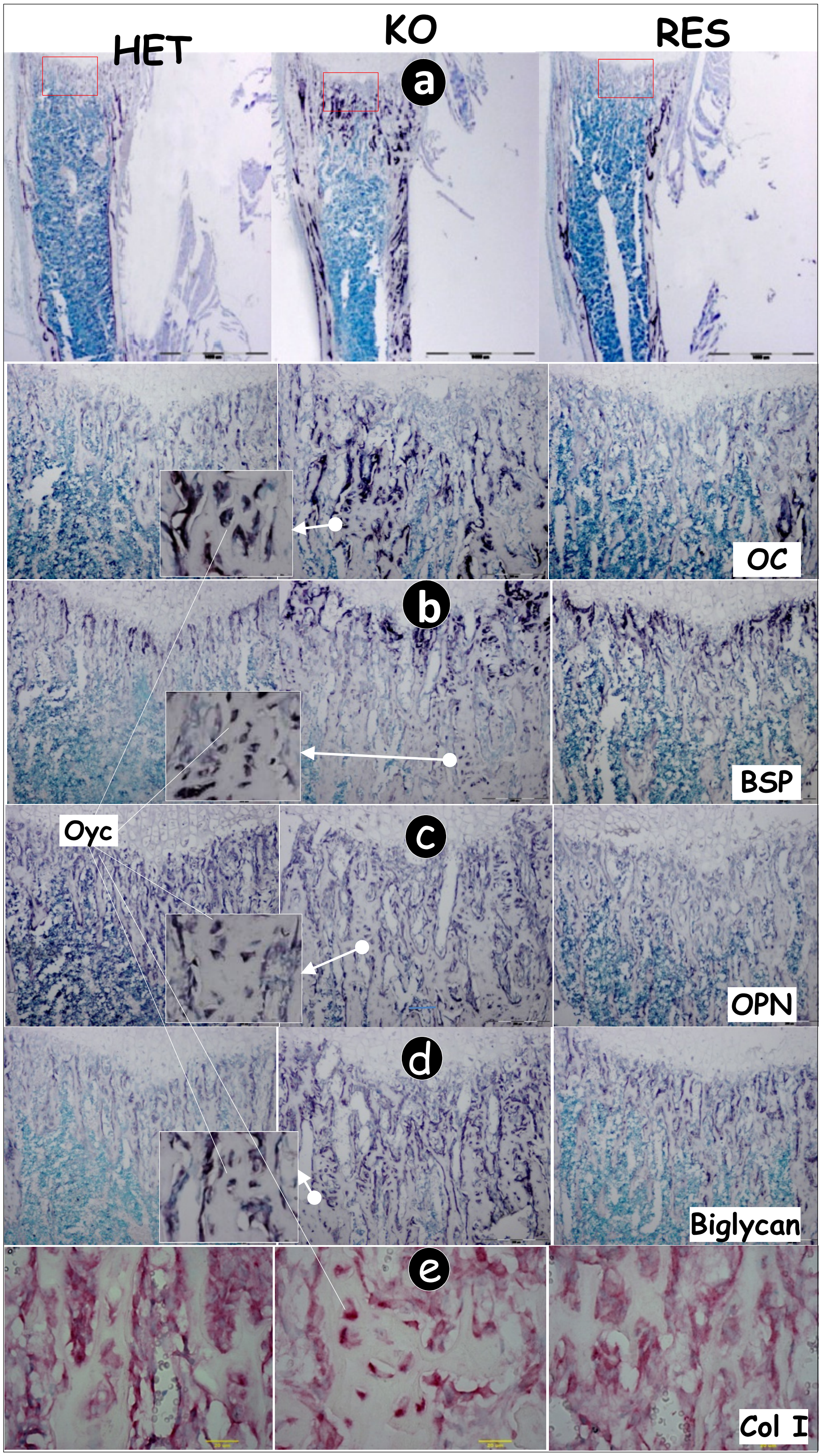

Fig S1. Targeted expression of DMP1 rescued the gene expression patterns in the *Dmp1*-null trabecular bone. The *in situ* hybridization (**a,** OC**; b,** BSP; **c,** OPN; **d,** Biglycan**;** and **e,** Col 1) assay was performed on the HET controls (*left panels*), the *Dmp1*-null (*middle panels*), and the RES (*right panels*) tibia trabeculae at age of 10 days. All these genes in *Dmp1*-null trabecular bone were increased (mainly in osteocytes) and restored to the control levels by the targeted expression of the full-length DMP1. Signal in dark purple color (a, b, c and d), and in red color (e).
